# Supplementary material for: Profiling non-small cell lung cancer reveals that PD-L1 is associated with wild type EGFR and vascular invasion, and immunohistochemistry quantification of PD-L1 correlates weakly with RT-qPCR
Source: PLoS One. 2021 May 6;16(5):e0251080. doi: 10.1371/journal.pone.0251080 (PMC8101740; doi:10.1371/journal.pone.0251080)
Supplement: S1 Table — (DOCX) [file pone.0251080.s001.docx]

S1 Table. List of primers used in the study.

| Primers | Nucleotide sequence (5’ → 3’) |
| --- | --- |
| PD-L1 | Forward: TATGGTGGTGCCGACTACAA  Reverse: TGCTTGTCCAGATGACTTCG |
| PD-1 | Forward: AAACTGGTACCGCATGAGCC Reverse: TTGTGTGACACGGAAGCGG |
| CD45 | Forward: CCTTCCCCCACTGGATTGAC Reverse: CTTTCAAAGGTGCTTGCGGG |
| CD8 | Forward: TTCTCGGGCAAGAGGTTGG Reverse: CAGGGCCGAGCAGAAATAGTA |
| CD3 | Forward: CACCTGTTCCCAACCCAGAC Reverse: AGATGCGTCTCTGATTCAGGC |
| TBP | Forward: GGCACCACTCCACTGTATCC  Reverse: GCTGCGGTACAATCCCAGAA |
| RPL13A | Forward: TTGGACTTTCCACCTGGTCATAT  Reverse: GTGTACAACAGCAAGCTCATGCT |
