# Supplementary material for: Profiling non-small cell lung cancer reveals that PD-L1 is associated with wild type EGFR and vascular invasion, and immunohistochemistry quantification of PD-L1 correlates weakly with RT-qPCR
Source: PLoS One. 2021 May 6;16(5):e0251080. doi: 10.1371/journal.pone.0251080 (PMC8101740; doi:10.1371/journal.pone.0251080)
Supplement: Attachment — Submitted filename: Response to reviewer 2021-03-31.doc [file pone.0251080.s003.doc]

6. Review Comments to the Author

Reviewer #1: Authors have appropriately replied to review comments. However, in discussion, authors described as below; "Indeed, two meta-analysis studies have reported lack of associations between PD-L1 presence and EGFR mutations in lung cancer patients [33, 34]." The reference number 34 reported that there were the association between PD-L1 expression and EGFR mutation status, so the above description is thought to be inappropriate.

*We modified the sentence as “Some studies have reported lack of associations between PD-L1 expression and EGFR status in lung cancer patients [33]” and removed reference [34] there. It is followed by “In this study we found that ….”. These two sentences are switched the order (comparing to the previous version) to make a more clear flow.*
